# Bayesian modelling of the effect of climate on malaria in Burundi

**DOI:** 10.1186/1475-2875-9-114

**Published:** 2010-04-29

**Authors:** Hermenegilde Nkurunziza, Albrecht Gebhardt, Jürgen Pilz

**Affiliations:** 1 Department of Mathematics, Institute of Applied Pedagogy, University of Burundi, Burundi; 2 Department of Statistics, University of Klagenfurt, Austria

## Abstract

**Background:**

In Burundi, malaria is a major public health issue in terms of both morbidity and mortality with around 2.5 million clinical cases and more than 15,000 deaths each year. It is the single main cause of mortality in pregnant women and children below five years of age. Due to the severe health and economic cost of malaria, there is still a growing need for methods that will help to understand the influencing factors. Several studies have been done on the subject yielding different results as which factors are most responsible for the increase in malaria. The purpose of this study has been to undertake a spatial/longitudinal statistical analysis to identify important climatic variables that influence malaria incidences in Burundi.

**Methods:**

This paper investigates the effects of climate on malaria in Burundi. For the period 1996-2007, real monthly data on both malaria epidemiology and climate in the area of Burundi are described and analysed. From this analysis, a mathematical model is derived and proposed to assess which variables significantly influence malaria incidences in Burundi. The proposed modelling is based on both generalized linear models (GLM) and generalized additive mixed models (GAMM). The modelling is fully Bayesian and inference is carried out by Markov Chain Monte Carlo (MCMC) techniques.

**Results:**

The results obtained from the proposed models are discussed and it is found that malaria incidence in a given month in Burundi is strongly positively associated with the minimum temperature of the previous month. In contrast, it is found that rainfall and maximum temperature in a given month have a possible negative effect on malaria incidence of the same month.

**Conclusions:**

This study has exploited available real monthly data on malaria and climate over 12 years in Burundi to derive and propose a regression modelling to assess climatic factors that are associated with monthly malaria incidence. The results obtained from the proposed models suggest a strong positive association between malaria incidence in a given month and the minimum temperature (night temperature) of the previous month. An open question is, therefore, how to cope with high temperatures at night.

## Background

In Burundi, malaria is a major public health issue in terms of both morbidity and mortality with around 2.5 million clinical cases and more than 15,000 deaths each year. During the last decade, for example, malaria cases increased from 550,000 cases in 1991 to 2.8 million in 2001 in a total population estimated at 7 million [1]. In 2001, Burundi was the country most affected by malaria in the world [2]. Malaria is the single main cause of mortality in pregnant women and children below five years of age. Malaria continues to ravage millions of rural Burundians, despite concerted efforts to reduce malaria mortality [3,4]. This is often attributed to a number of factors, including poverty, limited access to basic health care and specialized health facilities, the cost-sharing system, and the under-funding of the health sector by the government. Currently, the government allocates only 2% - 4% of its national budget towards supporting the health sector. The direct economic costs of malaria that result from treatment and from time away from work or school are enormous, but the overall economic impact of malaria is likely to be much more substantial than suggested by estimates of direct costs alone [5].

Due to the severe health and economic cost of malaria epidemics, there is still a growing need for methods that will help to understand the influencing factors, allow forecasting, early warning, so that more effective control measures may be implemented [6]. Several studies have been done on the subject yielding different results as which factors are most responsible for the increase in malaria. Epidemiology studies on malaria in Africa have identified, in general, an association between climate variables and malaria [7,8]. An association between climate variability and the epidemics of malaria has been identified in seven sites of East African highlands in Ethiopia, Kenya and Uganda [9], suggesting that climate variability had an important influence in initiating epidemics in the highlands of East Africa. There was a significant and positive influence of interactions between maximum temperature, minimum temperature and rainfall on malaria transmission. The work of Pemola and Jauhari [10] investigated the relationship between climate and malaria incidence using Pearson's correlation analysis. The authors found a high positive correlation between monthly parasite incidence and climatic variables (temperature, rainfall and humidity). Gallup and Sachs [11] have suggested that the location and severity of malaria are mostly determined by climate and ecology. A significant correlation between malaria risk and elevation, annual maximum temperature and rainfall was also described [12]. Another study found that variation in the malaria transmission intensity was strongly associated with basic climatic factors, noting that even small differences in climate variation can significantly affect malaria transmission intensities [13]. Rainfall, temperature and altitude were the most plausible predictors of malaria prevalence in Botswana [14].

Others have suggested that malaria was influenced by factors other than climate. Hay *et al *[15] suggested that the association between local malaria resurgence and regional changes in climate was overly simplistic. Economic, social and political factors may explain recent resurgence in malaria and other mosquito-borne diseases, with no need to invoke climatic changes. Many variables may affect malaria transmission beside climatic changes, such as environmental modification, population growth, limited access to health care, and lack of or unsuccessful malaria control measures [16]. Although climate can affect the incidence of malaria, human economic activities and malaria control strategies play an important role in the incidence of the disease [17]. Relatively high rates of malaria morbidity could result from poor access to health services, inadequate case management, overwhelmed health services, poor immunological competence because of malnutrition, a general disruption to livelihoods because of often-associated flooding, or a combination of these factors [18].

The above-mentioned studies have led to various controversial conclusions when investigating which factors influence malaria incidence, depending on the country specificity. This makes it difficult to establish common determinants of malaria in all countries [19]. In Burundi, the situation of malaria is poorly documented. Indeed, very few studies have been published on the malaria prevalence in Burundi. Further, published papers concern only three provinces (Ngozi, Kayanza, Karuzi) [20,21], where "Médecins-sans-Frontières" from Belgium was active. It is believed that the situation of crisis experienced by Burundi since 1993, followed by a break in cooperation with some countries led researchers and international agencies to lose interest on Burundi. So far, no research on malaria has been carried out throughout the entire country in order to establish a generalized framework to investigate this subject.

In this paper, both generalized linear and generalized additive mixed models are proposed to assess the climatic factors that are the highly associated with monthly malaria incidence in Burundi. It is assumed that malaria incidence in a given month in Burundi can be predicted by rainfall, temperature and humidity. Rainfall has a great influence on the mosquito population by increasing the vegetation density and by providing suitable breeding pools for the production and maturation of larvae. Temperature has a great influence on the transmission of malaria, higher temperatures shortening the extrinsic incubation period [6] and humidity facilitates adult mosquito life span [9,22,23].

## Methods

### Study area

Burundi is located in East-central Africa, between 2°20 and 4°27 of latitude south and between 28°50 and 30°53 of longitude east; the altitude varies between 775 metres (Lake Tanganyika) and 2,670 metres (Crest Congo - Nil). Burundi has generally a tropical highland climate, with a considerable daily temperature variation in many areas [24]. Temperature also varies considerably from one region to another, mainly as a result of differences in altitude. The central plateau is cool, with temperature averaging 20°C. The area near Lake Tanganyika is warmer, averaging 23°C; the highest mountain areas are cooler, averaging 16°C. Rain is irregular, falling most heavily in the north-west [24]. Dry season varies in length, and there are sometimes longer periods of drought. Most parts of Burundi receive between 130 and 160 cm of rainfall a year [24]. Bounded on the north by Rwanda, in south-east by Tanzania and in west by the Democratic Republic of Congo, Burundi covers an area of 27,834 km^2 ^(of which 2,634 km^2 ^are occupied by Tanganyika Lake) and has a population estimated at about 8 million. In terms of habitat, it remains essentially rural, with 91.6% of the population living in rural areas. The urban population is 8.4% with an annual growth rate of 5.7%. The Burundi population is young: 46.1% are under 15 years of age, while people aged 60 and above represent only 5.4%. With an average density of 266 inhabitants per km^2^, a population growth rate of 3.44% and a total fertility rate of six children per woman, Burundi is one of Africa's most densely populated country [3]. Burundi is structured in 17 provinces. The epidemiological profile can be summarized as follows. The health system suffers from a shortage of qualified personnel with one doctor per 34,750 inhabitants and one nurse for 3,500 inhabitants. 17.4% of patients do not have access to health care, while 81.5% of patients are forced to go into debt or sell property to pay the health costs. There is a big disparity between the capital Bujumbura and the remainder of the country, as 80% of doctors and more than 50% of nurses are working in Bujumbura. Malaria, which is responsible for more than 50% of hospital deaths in children under five years of age and 40% of all consultations in health centres, is undoubtedly the main public health problem, the main cause of mortality and morbidity in Burundi [3].

### Data description

#### Malaria data

The goal/aim of this study is to assess which climatic factors affect malaria incidence in Burundi. Data on malaria morbidity in Burundi were collected from EPISTAT (Epidemiology and Statistics) [25], a department of the Burundi Ministry of health, in charge of collecting and storing data on epidemiology all over the country. The health services collect all monthly notification of malaria consultations. Malaria morbidity data/number from 1996 to 2007 were collected. This is the period where data are available in EPISTAT. The well-known nearest neighbour method is used to fill the (~5%) missing data. The estimated population for each province, for the study period, was obtained from the Institute of Statistics and Economic Studies of Burundi (ISTEEBU) [26]. In this study, the number of malaria cases for each province was divided by the total number of population of the province to obtain the incidence rate. Used are the incidence rates per 1,000 inhabitants.

#### Meteorological data

Monthly cumulative precipitation, monthly average of maximum temperature, monthly average of minimum temperature, monthly average of maximum humidity and monthly average of minimum humidity, for the period 1996-2007 were obtained from the Geographic Institute of Burundi (IGEBU) [27]. The record of these variables from 1996 to 2007 has remained uniform, with the same calibration and the same precision. Only 14 stations had enough data for the desired study period. The missing data (2% - 3%) were filled by the same method as in Malaria data (nearest neighbour and cross-validation). Data for three provinces (Bubanza, Bujumbura rural and Cibitoke) were not available for the study period. They are estimated using ordinary kriging [28].

### Modelling the impact of climatic variables on malaria

#### Generalized linear and generalized additive mixed models

Let _*Y *_be a response variable, *X *= (*X*_1_, ..., *X*_*n*_) be a vector of covariates and *θ *and *ϕ *unknown parameters. Bayesian generalized linear models assume that the distribution of *Y *belongs to an exponential family, i.e.(1)

Here *b*(.), *c*(.), *θ *and *ϕ *determine the specific response distribution [29-31]. The mean *μ *= *E*(*Y*/*X*, *γ*) is linked to a linear predictor *η *by(2)

where *h *is a known link function and *γ *is a vector of unknown regression parameters.

In most practical regression applications however, the assumption of a strictly linear effect (of the covariates) on the predictor may not be appropriate for continuous covariates.

Generalized additive mixed models for longitudinal data generalize (2), by replacing the strictly linear predictor by a structured additive predictor [32]:(3)

where *η*_*it *_is the predictor, *x*_*it*1_, ..., *x*_*itk*_, *u*_*it *_are the (continuous) covariates values observed for location *i *at time *t*, *f*_*j *_are smooth function and *ε*_*it *_is the error. For Bayesian inference, the unknown functions *f*_1_, ...., *f*_*p *_in the predictor (3) and the fixed effects parameter *γ *are onsidered as random variables and are supplemented by appropriate prior assumptions [33]. Defining the vector of the function evaluations *f*_*j *_= (*f*_*j*_(*x*_*it*1_), ..., *f*_*j*_(*x*_*itp*_))' as the matrix product of a design matrix *X*_*j *_and a vector of unknown parameters *β*_*j*_, i.e. *f*_*j *_= *X*_*j*_*β*_*j*_, one obtains the predictor (3) in matrix notation as(4)

where *U *corresponds to the design matrix for fixed effects. A prior for a function *f*_*j *_is defined by specifying a suitable design matrix *X*_*j *_and a prior distribution for the vector *β*_*j *_of unknown parameters. The general form of the prior for the *β*_*j *_in (4) is given by(5)

where *K*_*j *_is a penalty matrix that shrinks parameters towards zero or penalizes too abrupt jumps between neighbouring parameters [32,34]. The variance parameter  in (5) controls the trade-off between flexibility and smoothness. Weakly informative inverse Gamma hyperpriors ~*IG*(*a*_*j*_, *b*_*j*_) are often assigned to . Bayesian inference is based on the posterior of the model given by:(6)

Here *L*(.) denotes the likelihood which, under the assumption of conditional independence, is the product of individual likelihood contributions. Markov Chain Monte Carlo (MCMC) simulation methods that allow to draw random samples from the posterior are used to compute the posterior distribution [35,36].

### Model formulation

The aim is to model the dependence of malaria incidence on covariates including rainfall, minimum and maximum temperature, minimum and maximum humidity in Burundi. In the model, the variation of temperature, rainfall and humidity within one province is assumed not significant. This assumption is dictated by the fact that provinces in Burundi are small (17 provinces on an area of 27,834 km^2^). Taking into account the life cycle of the parasite (13 days for *Plasmodium falciparum*) [13] and the incubation period (seven days to four weeks), it is assumed that malaria incidence in a given month is associated with climatic conditions of the same month and those of the previous month. Most of those who become ill in a given month were bitten by mosquitoes in the previous month. The data are available in different scale and units (malaria and humidity data are unit-free, rainfall is measured in centimetres (cm) and temperature in degrees centigrade (°c)). To avoid the effect of scale in our modelling, the data are first standardized. Throughout this study we adopt the following notation for the variables:*R*_*n *_is the rainfall, *T*_*x *_is the maximum temperature, *T*_*n *_is the minimum temperature, *H*_*x *_is the maximum humidity, *H*_*n *_is the minimum humidity and *R*_*np*_, *T*_*xp*_, *T*_*np*_, *H*_*xp*_, *H*_*np *_are the same variables for the previous month.

**Figure 1:**
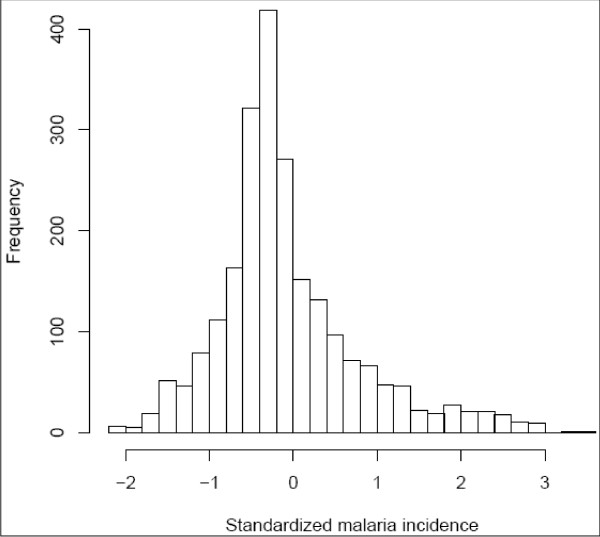
**Histogram of the standardized malaria incidences data**.

Figure 1 represents the histogram of the standardized malaria incidences. The histogram is right-skewed, suggesting that a gamma distribution for the response variable is appropriate. Since some values of the standardized data are negative, a second transformation is made to the data by adding a constant (*c *= 2.2) to the response variable to obtain positive values. The following analysis is then conducted on these new data. In the model, the response variable *Y*(malaria incidence) is assumed to have a gamma distribution (with log-link function), i.e.(7)

where *μ *> 0 is the mean and *s *> 0 is the shape parameter. The parameters *μ *and *s *are related to *θ *and *ϕ *in equation (1) by  and  (see [32] for more details).

### Generalized linear model

The following generalized linear model is first fitted using all the available covariates.(8)

Here

*η*_*it *_is the predictor of malaria incidence assumed to have a gamma distribution,

*Y*_*it *_is the malaria incidence in province *i *(*i *= 1,...,17) and month *t*(*t *= 1,...,144),

*β *is a vector of unknown regression parameters *β*_*k*_,

*X*_*kit *_and *X*_*kip *_are the five climatic covariates (rainfall, maximum temperature, minimum temperature, maximum humidity and minimum humidity) for months *t *and the previous month *p *respectively. Finally, *β*_0 _is the intercept (accounting for the effects of unmeasured covariates) and *ε*_*it *_is the error. Variables selection is then performed based on the Akaike information criterion (AIC) in a stepwise algorithm; a final model was then retained. The lowest AIC corresponded to the following model:(9)

The regression coefficients *β*_*k *_(*k *= 0,...,5) are estimated via Markov Chain Monte Carlo (MCMC) simulation that allows to draw random samples from the posterior given by (6).

12,000 iterations of the MCMC are ran with a burn-in phase of 2,000 iterations. A thinning to the Markov Chain is applied to reduce autocorrelations, by requiring the programme to store only every 10^th ^sampled parameter. This leads to a random sample of length 1,000 for every parameter in the model [35].

The estimated values of the parameters *β*_*i *_are presented in Table 1.

**Table 1:** Estimates of the coefficients of the linear effects in the generalized linear model.

Parameter	Mean	**Std. Dev**.	Median	95% Confidence Interval
*β*_0_	0.751051	0.0083366	0.750995	[0.734, 0.767]

*β*_1_	-0.028041	0.0099073	-0.028237	[-0.047, -0.008]

*β*_2_	-0.043121	0.0122528	-0.042746	[-0.067, -0.019]

*β*_3_	-0.016150	0.0109984	-0.016187	[-0.038, 0.004]

*β*_4_	0.047941	0.00983923	0.048019	[0.029, 0.066]

### Generalized additive mixed model

Assuming a nonlinear effect of the climatic covariates on malaria incidence, a generalized additive model (GAM) is first fitted tothe longitudinal data. The additive approximation has the following advantages: first, the curse of dimensionality is avoided because each of the individual additive terms is estimated using a univariate smoother [37]. Second, the estimates of the individual terms explain how the dependent variable changes with the corresponding independent variables [37]. At the beginning, all the explanatory covariates are used as follows;(10)

Here, as above, *η*_*it *_is the predictor of malaria incidence in month *t *and province *i*, assumed to have a gamma distribution, *X*_*kit *_and *X*_*kip *_are the five covariates in province *i *for month *t *and the previous month *p*, respectively, *f*_*i *_are unknown smooth functions of the covariates, *β*_0 _is the intercept and *ε*_*it *_is the error. The effects of two-factors interaction are assumed to be smaller and are consequently omitted. The main reason is the wish to preserve the simplicity and easy interpretation of the effects, which are often lost by including interactions [29,38].

A final model was then selected based on the Akaike information criterion (AIC) using the algorithm described in [39]. The algorithm is able to decide whether a particular covariate is included in the model or not and whether it enters the model linearly or nonlinearly. Moreover the algorithm selects an appropriate degree of smoothness of the nonlinear covariate. Model choice and estimation of the parameters is done simultaneously (see [39] for more details). The lowest AIC corresponded to the following generalized additive mixed model (GAMM) [32]:(11)

The regression coefficients *α*_*k *_(*k *= 0,...,3) and the nonlinear effect of the continuous covariates are estimated via Markov Chain Monte Carlo (MCMC) as above. The effects of the continuous covariates are modelled by cubic p-splines [40] with 20 equidistant knots and second order random walk penalty [38,41]. Positive hyperparameters *a *= 0.001 and *b *= 0.0005 for *τ *^2 ^are chosen to ensure the propriety of the posterior [35,40,42]. The sensitivity of the results with respect to changes in the hyperparameters *a *and *b *is then checked by re-estimating the model with different choices for the hyperparameters *a *and *b *for each effect in the model by setting

(*a *= 1, *b *= 0.005); (*a *= 0.001, *b *= 0.001); (*a *= 0.01, *b *= 0.005); (*a *= 0.001, *b *= 0.005) (*a *= 0.0001, *b *= 0.0001); (*a *= 0.0001, *b *= 0.0005) to assess the dependence of results on minor changes in the model assumptions. The results showed no significant change.

The models are implemented in BayesX, a public domain software for Bayesian inference in structured Additive Regression Models available at http://www.stat.uni-muenchen.de/~bayesx/bayesx.html.

## Results and discussion

The purpose of this study has been to undertake a spatial/longitudinal statistical analysis to identify important climatic variables that influence malaria incidences in Burundi. Table 1 presents the estimates of the coefficients *β*_*i *_from model (9).

In Table 1, *β*_1_, *β*_2 _and *β*_3 _have negative means. Moreover *β*_1 _and *β*_2 _have a negative 95% credible interval (CI), suggesting that rainfall and maximum temperature in a given month have a negative effect on malaria incidence of the same month. *β*_4 _has a positive mean with a positive 95% CI. This suggests malaria incidence in a given month is positively and significantly associated with minimum temperature of the previous month.

**Figure 2:**
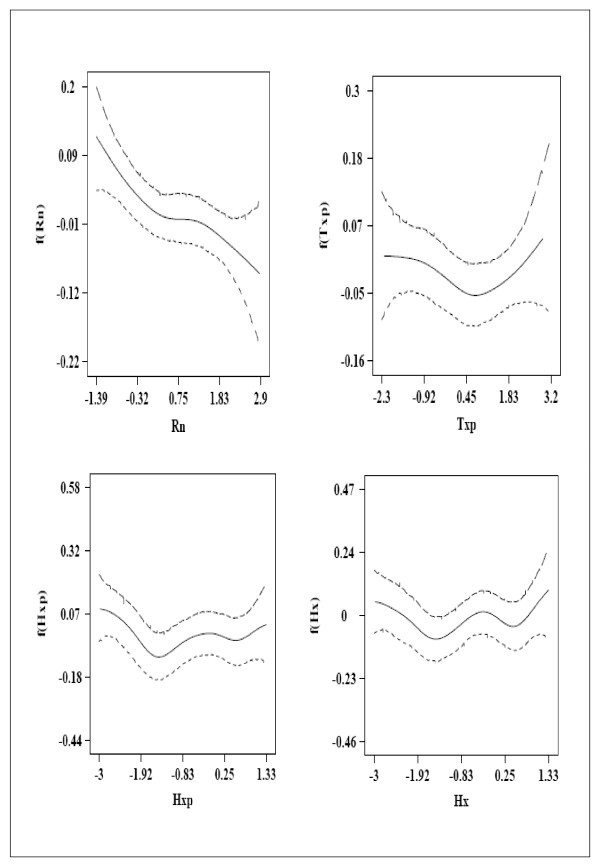
**Estimates of the nonlinear effects of the continuous covariates in model (11) with 95% CI**.

The coefficients *α*_*i *_of the linear part of model (11) are given in Table 2: *α*_1 _has negative mean with a negative 95% CI. This suggests that maximum temperature in a given month has a negative effect on malaria incidence of the same month. *α*_2 _and *α*_3 _have positive means, moreover *α*_3 _has a positive 95% CI. This suggests that malaria incidence in a given month is positively associated with the minimum temperature of the same month and (more significantly) that of the previous month. The results of the GAMM are in good agreement with those of the GLM. In both tables the intercepts (*β*_0 _and *α*_0_) have positive mean with a positive 95% CI. Moreover, they have the largest absolute value. This suggests that variables other than climate may have a greater influence on malaria incidence. The extents of nonlinear effects are represented in Figure 2. The upper left plot of Figure 2 suggests that rainfall in a given month has a negative effect on malaria incidence of the same month. Other plots show no clear trend.

**Table 2:** Estimated coefficients of the linear effects in the generalized additive mixed model.

Parameter	Mean	**Std. Dev**.	Median Interval	95% Confidence
*α*_0_	0.781362	0.0520609	0.780101	[0.677, 0.884]

*α*_1_	-0.0381613	0.0134649	-0.0380738	[-0.064, -0.0109]

*α*_2_	0.0155423	0.0151579	0.0157556	[-0.014, 0.044]

*α*_3_	0.035133	0.0142763	0.0347205	[0.0064, 0.063]

Overall, both models (GLM and GAMM) suggest that malaria incidence in a given month in Burundi is significantly positively associated with the minimum temperature of the previous month. Conversely, rainfall and maximum temperature in a given month are found to have a negative effect on malaria incidence of the same month. Possible explanations of these results are the following. Due to the development cycle of the parasite into mosquitoes and the incubation period, those who became ill in a given month were bitten by mosquitoes in the previous month. Minimum temperature is observed at night and mosquitoes are active only at night; by day-time they hide themselves in houses or vegetation. This explains why this factor, which is observed at the same time (at night) when there is mosquito activity, has a great influence on malaria transmission. Moreover, when the night temperature is high, people do not cover themselves, increasing the risk of being bitten by mosquitoes. Maximum temperature has a negative effect because mosquito's development is interrupted at higher temperature [41]. Too much rainfall may flush away breeding larvae, reducing the numbers of the disease vectors.

## Conclusions

This study has exploited available real monthly data on malaria, rainfall, temperature and humidity over 12 years (1996-2007) in the area of Burundi to derive and propose a regression modelling to assess climatic factors that are associated with monthly malaria incidence. The regression is based on the generalized linear model (GLM) and generalized additive mixed model (GAMM). The results obtained suggest a strong positive association between malaria incidence in a given month and the minimum temperature (night temperature) of the previous month. An open question is therefore how to cope with high temperatures at night. One possible suggestion in the context of Burundi would be to popularize the use of bed nets in order to reduce contact with mosquitoes. In contrast, it is found that rainfall and maximum temperature in a given month have a negative effect on malaria incidence of the same month. It is believed that climatic variables alone cannot explain the spatio-temporal distribution of malaria cases in Burundi. An interesting problem under investigation is extending the results in this work by incorporating in the proposed model the spatial effect by accounting for both structured (correlated) and unstructured (uncorrelated) components.

## Competing interests

The authors declare that they have no competing interests.

## Authors' contributions

HN collected the data, performed the statistical analysis and drafted the manuscript. AG and JP reviewed and improved the manuscript. All authors read and approved the final manuscript.
